# Artificial Intelligence Literacy, Critical Thinking Disposition, and Clinical Competence Among Nursing Students: A Cross-Sectional Study

**DOI:** 10.3390/nursrep16080281

**Published:** 2026-08-12

**Authors:** Yeowon Jeong, Nayoung Kim, Seongha Park, Jiyun Seok

**Affiliations:** Department of Nursing, Daegu Haany University, Gyeongsan 38610, Republic of Korea; swan11150@dhu.ac.kr (Y.J.); psh030909@dhu.ac.kr (S.P.); sjotjrwldbs@dhu.ac.kr (J.S.)

**Keywords:** nursing, AI literacy, critical thinking disposition, clinical competence

## Abstract

**Highlights:**

**What are the main findings?**

- AI literacy and critical thinking disposition were significantly associated with clinical competence among nursing students with clinical practice experience.
- Satisfaction with clinical practice, critical thinking disposition, AI literacy, and academic year were identified as significant factors associated with clinical competence.

**What are the implications of the main findings?**

- Nursing education may consider AI literacy as an emerging educational competency associated with clinical competence in rapidly changing healthcare environments.
- AI literacy and critical thinking disposition may be considered together in nursing education strategies related to clinical competence.

**Abstract:**

**Background/Objectives**: As artificial intelligence (AI) becomes increasingly integrated into healthcare, AI literacy and critical thinking disposition are recognized as important competencies for nursing students in rapidly changing clinical environments. This study aimed to examine the associations of AI literacy and critical thinking disposition with clinical competence among nursing students. **Methods**: A descriptive cross-sectional study was conducted with 200 nursing students with clinical practice experience who were enrolled in nine nursing colleges located in D City and K Province, Republic of Korea. Data were collected using a structured questionnaire and analyzed using IBM SPSS/WIN 25.0. Statistical analyses included descriptive statistics, *t*-tests, analysis of variance (ANOVA), Scheffé post hoc tests, Pearson’s correlation coefficients, and multiple regression analysis. **Results**: Satisfaction with clinical practice (β = 0.35, *p* < 0.001), critical thinking disposition (β = 0.34, *p* < 0.001), AI literacy (β = 0.23, *p* = 0.001), and academic year (β = 0.15, *p* = 0.009) were significantly associated with clinical competence. The model explained 40% of the variance in clinical competence (R^2^ = 0.40, adjusted R^2^ = 0.39, F = 26.11, *p* < 0.001). **Conclusions**: The findings indicate that critical thinking disposition and AI literacy were significantly associated with clinical competence among nursing students. Nursing education may therefore consider strategies that address both competencies.

## 1. Introduction

Recent advances in artificial intelligence (AI) have transformed multiple sectors, including education and healthcare. As AI becomes increasingly integrated into healthcare, AI literacy has gained recognition as an essential competency for preparing nurses to critically understand, evaluate, and responsibly use AI technologies in clinical settings [1,2]. The integration of AI into nursing practice has the potential to support clinical decision-making, facilitate the early detection of patient deterioration and risks, improve workflow efficiency, and enhance patient safety and clinical outcomes [3,4]. However, concerns remain regarding the accuracy and reliability of AI-generated information, limited transparency and explainability, excessive reliance on automated systems, and risks to data privacy and confidentiality [5,6]. These benefits and risks underscore the need for nurses to develop the competencies required to integrate AI into practice safely, critically, and responsibly.

These developments underscore the importance of preparing nursing students to understand and appropriately use AI technologies. AI literacy is defined as a set of competencies that enable individuals to critically evaluate AI technologies, communicate and collaborate effectively with AI, and use AI as a tool online, at home, and in the workplace [7]. The scope of AI literacy can be more clearly understood by considering its relationship with digital literacy, nursing informatics competencies, and digital self-efficacy. Digital literacy refers to the ability to access, manage, understand, integrate, communicate, evaluate, and create information safely and appropriately through digital technologies [8]. Nursing informatics competencies encompass skills beyond those related to computers, as well as the knowledge and attitudes needed by nurses [9]. Digital self-efficacy refers to an individual’s self-efficacy regarding the effective and effortless use of information technology and adaptation to updates in hardware and software [10]. Meanwhile, AI literacy concerns the competencies required to understand, evaluate, and use AI technologies and has been conceptualized as a multidimensional construct encompassing knowledge and understanding of AI, application, critical evaluation, and ethical considerations [7,11,12]. In this study, AI literacy was defined in terms of five dimensions: AI knowledge, AI utilization, AI impact analysis, AI problem-solving capability, and AI ethical thinking [13].

Critical thinking disposition refers to the consistent internal motivation to engage with problems and make decisions by using critical thinking [14]. Because clinical practice requires nurses to make judgments and solve problems based on critical thinking [15], critical thinking disposition may be relevant to clinical competence. Studies involving nursing students have reported positive associations between critical thinking disposition and clinical competence [16,17]. Related findings have also been reported among clinical nurses in Iran and Taiwan, although the study populations and the critical thinking constructs examined differed from those of the present study [18,19].

The need for critical thinking has become even more pronounced as AI-generated information is increasingly used in nursing education and clinical settings. Appropriate use of AI-generated information requires learners to question its accuracy and credibility, recognize potential limitations and biases, and determine whether it is suitable for a specific clinical context [1]. A multicenter cross-sectional study of nursing interns found that AI literacy was positively associated with critical thinking ability and partially mediated the association between AI dependence and critical thinking ability [20]. Although the concepts examined were not directly equivalent, a study of registered nurses also found a significant positive correlation between nursing informatics competencies and the critical thinking dimension of clinical decision-making [21]. These findings suggest that both AI literacy and critical thinking may warrant consideration in nursing education, particularly as students are increasingly required to evaluate AI-generated information in clinical contexts.

Clinical competence refers to the ability to effectively perform a range of tasks in clinical settings by applying nursing knowledge, judgment, skills, and attitudes [22]. Nurses with high levels of clinical competence are able to respond promptly to changes in patients’ conditions and to demonstrate effective communication and problem-solving abilities, thereby delivering high-quality nursing care even in complex healthcare environments [23]. For nursing students, the attainment of clinical competence indicates that they have reached a level at which they can demonstrate the professional behaviors expected of nurses [24], and it is therefore regarded as an important outcome indicator in nursing education. Although direct empirical evidence on the relationship between AI literacy and clinical competence remains limited, previous studies provide indirect evidence regarding specific competencies encompassed by AI literacy. In particular, the ability to critically evaluate AI-generated information may help nursing students assess its accuracy and clinical appropriateness rather than accept it uncritically [25]. Ethical awareness regarding AI may also promote consideration of privacy, accountability, fairness, transparency, and patient safety [26,27]. These critical evaluation and ethical dimensions of AI literacy may be relevant to clinical reasoning and decision-making by promoting the appropriate use of AI-generated information while maintaining nurses’ professional judgment and responsibility [26,27,28].

Accordingly, AI literacy and critical thinking disposition may each be associated with clinical competence and may also be related to one another. These potential relationships support the concurrent examination of AI literacy and critical thinking disposition in relation to clinical competence among nursing students.

Recent international research involving nursing students has largely focused on attitudes and perceptions toward AI, readiness for AI use, technology acceptance or adoption intentions, and perceived barriers or concerns related to its implementation [29,30,31]. However, relatively little attention has been given to the relevance of AI literacy to core nursing education outcomes. In particular, empirical evidence regarding the relationship between AI literacy and clinical competence remains scarce, and research examining AI literacy and critical thinking disposition concurrently in relation to clinical competence remains limited. In addition to these core variables, general characteristics may also be associated with clinical competence. Previous studies have reported that academic year and satisfaction with clinical practice are associated with clinical competence among nursing students [32,33]. General and AI use-related characteristics were therefore examined on an exploratory basis in the present study.

This study therefore aimed to assess the levels of AI literacy, critical thinking disposition, and clinical competence among nursing students with clinical practice experience and to identify factors associated with clinical competence. The findings may provide preliminary evidence regarding the educational relevance of AI literacy and inform future nursing education strategies that support the critical, appropriate, and responsible use of AI in clinical practice.

The specific objectives of this study were as follows: first, to assess the levels of AI literacy, critical thinking disposition, and clinical competence among the participants; second, to examine differences in clinical competence according to the participants’ general characteristics; third, to investigate the correlations among AI literacy, critical thinking disposition, and clinical competence; and fourth, to identify the factors associated with the participants’ clinical competence.

## 2. Materials and Methods

### 2.1. Study Design

This study used a descriptive cross-sectional survey design to examine AI literacy and critical thinking disposition as factors associated with clinical competence among nursing students with clinical practice experience in the Republic of Korea.

### 2.2. Participants

Participants were recruited through convenience sampling from nine nursing colleges located in D City and K Province, Republic of Korea. The inclusion criteria were as follows: (1) nursing students with clinical practice experience, (2) ability to understand and complete the questionnaire, (3) adequate understanding of the purpose and nature of the study, and (4) voluntary agreement to participate.

The required sample size was calculated using G*Power version 3.1.9.7 (Heinrich-Heine-Universität Düsseldorf, Düsseldorf, Germany). For multiple regression analysis, assuming a significance level of 0.05, a statistical power of 95%, a medium effect size of 0.15, and 10 independent variables, the minimum required sample size was determined to be 172. Considering an anticipated rate of incomplete responses of approximately 15–20%, 202 nursing students were recruited. After two incomplete or unreliable responses were excluded, data from 200 participants were included in the final analysis.

### 2.3. Measures

#### 2.3.1. AI Literacy

In this study, AI literacy was measured using the AI Literacy Scale developed by Kang [13] for undergraduate and graduate students. The scale was developed based on a multidimensional conceptualization of AI literacy derived from a literature review, in-depth interviews with AI experts, and topic modeling of social media data from global AI experts. Content validity was evaluated by eight experts, and face validity was assessed through a pilot study involving 42 undergraduate and graduate students. Construct validity was examined using exploratory and confirmatory factor analyses with data from 400 participants, together with assessments of convergent and discriminant validity. The final instrument consists of 19 items across five subdomains: AI utilization (5 items), AI impact analysis (4 items), AI problem-solving capability (3 items), AI knowledge (4 items), and AI ethical thinking (3 items). Each item is rated on a 5-point Likert scale ranging from 1 to 5, with higher scores indicating higher levels of AI literacy. Cronbach’s α was 0.91 in the original study, 0.90 in a previous study involving nursing students [34], and 0.89 in the present study.

#### 2.3.2. Critical Thinking Disposition

In this study, critical thinking disposition was assessed using the instrument developed by Yoon [35] for nursing students and nurses. The instrument comprises 27 items across seven subdomains: intellectual eagerness/curiosity (5 items), prudence (4 items), self-confidence (4 items), systematicity (3 items), intellectual fairness (4 items), sound skepticism (4 items), and objectivity (3 items). Each item is rated on a 5-point Likert scale ranging from 1 to 5, with higher scores indicating a higher level of critical thinking disposition. In the original study, Cronbach’s α was 0.84, and in the present study, Cronbach’s α was 0.89.

#### 2.3.3. Clinical Competence

In this study, clinical competence was measured using the instrument originally developed by Lee [36] and later revised and supplemented by Choi [37]. The instrument consists of 45 items across five subdomains: nursing process (11 items), nursing skills (11 items), education/collaborative relationships (8 items), interpersonal relationships/communication (6 items), and professional development (9 items). Each item is rated on a 5-point Likert scale ranging from 1 to 5, with higher scores indicating higher levels of clinical competence. In the original study, Cronbach’s α was 0.92. In Choi’s study [37], Cronbach’s α was 0.92, and in the present study, Cronbach’s α was 0.94.

### 2.4. Data Collection and Procedure

In this study, data were collected using a self-report questionnaire administered in both paper-based and online formats. The survey was conducted from 4 September to 12 November 2025. Prior to participation, the purpose of the study and the eligibility criteria were explained verbally, and written informed consent was obtained from those who expressed willingness to participate. For the online survey, participants were provided with a survey link that included information about the study purpose and procedures, and they were allowed to proceed only after providing voluntary consent. Participants received a small token of appreciation after completing the survey.

### 2.5. Ethical Considerations

This study was conducted after obtaining approval from the Institutional Review Board (IRB) of Daegu Haany University (IRB No. 2025213). To minimize any possibility of coercion, student researchers who had no role in teaching, grading, or evaluating the participants assisted with recruitment and data collection. Participants were informed that their participation was entirely voluntary, that they could withdraw from the study at any time without any disadvantage, and that refusal to participate or withdrawal from the study would not affect their grades, academic standing, or relationship with the university. The collected data were used solely for the purposes of this study, and all data were anonymized and de-identified by assigning codes instead of personal identifiers to prevent the identification of individual participants. Hard-copy materials were stored in a double-locked cabinet and will be retained for 3 years after study completion, after which they will be destroyed. Electronic data were stored in an encrypted folder on the researcher’s password-protected personal computer and will be permanently deleted using a nonrecoverable method.

### 2.6. Data Analysis

The collected data were analyzed using IBM SPSS Statistics version 25.0 (IBM Corp., Armonk, NY, USA). The specific analytical procedures were as follows. First, the participants’ general characteristics were analyzed using frequencies, percentages, means, and standard deviations. Differences in clinical competence according to the participants’ general characteristics were examined using the independent *t*-test and analysis of variance (ANOVA), and post hoc comparisons were conducted using the Scheffé test. Second, the levels of AI literacy, critical thinking disposition, and clinical competence were analyzed using means and standard deviations. Third, the correlations among AI literacy, critical thinking disposition, and clinical competence were examined using Pearson’s correlation coefficient. Fourth, multiple regression analysis was conducted to identify factors associated with clinical competence.

## 3. Results

### 3.1. General Characteristics

The general characteristics of the participants are shown in Table 1. The average age of the participants was 22.18 years, and the gender distribution was 83.5% female and 16.5% male. In terms of academic year, 56.0% were third-year students and 44.0% were fourth-year students. Regarding satisfaction with clinical practice, the largest proportion of students reported “moderate” satisfaction (44.5%), followed by “satisfied” (43.0%) and “dissatisfied” (12.5%). Regarding academic performance, 29.0% of students reported “high” performance, 53.5% reported “moderate,” and 17.5% reported “low.” Regarding the frequency of generative AI use, most participants reported using generative AI frequently (63.0%), followed by those who used it only when necessary (35.0%). Regarding the purpose of generative AI use, the most common purpose was assignments and studying (72.0%), followed by information retrieval (15.5%). Regarding perceived reliance on generative AI, 58.5% of participants reported high reliance, while 35.0% reported moderate reliance.

### 3.2. Differences in Clinical Competence by General Characteristics

Clinical competence differed significantly according to academic year (t = −3.02, *p* = 0.003) and satisfaction with clinical practice (F = 14.86, *p* < 0.001) (Table 1). The average score for fourth-year students was significantly higher than that of third-year students. Scheffé post hoc analysis showed that students who were satisfied with clinical practice had higher clinical competence scores than those with moderate satisfaction, and those with moderate satisfaction scored higher than those who were dissatisfied.

### 3.3. Levels of AI Literacy, Critical Thinking Disposition, and Clinical Competence

The participants’ AI literacy, critical thinking disposition, and clinical competence are shown in Table 2. The participants’ AI literacy scores were 65.51 ± 10.69 out of 95, their critical thinking disposition scores were 100.98 ± 11.23 out of 135, and their clinical competence scores were 179.19 ± 23.10 out of 225.

### 3.4. Correlations Among AI Literacy, Critical Thinking Disposition, and Clinical Competence

The results of the analysis of correlations among the participants’ AI literacy, critical thinking disposition, and clinical competence are shown in Table 3. AI literacy was positively correlated with critical thinking disposition (*r* = 0.59, *p* < 0.001) and clinical competence (*r* = 0.48, *p* < 0.001), and critical thinking disposition was positively correlated with clinical competence (*r* = 0.51, *p* < 0.001).

### 3.5. Factors Associated with Clinical Competence

Multiple regression analysis was conducted to identify variables associated with the clinical competence of nursing students with clinical practice experience. AI literacy and critical thinking disposition were included as key independent variables. Among the general characteristics, academic year and satisfaction with clinical practice were entered into the model because they showed significant differences in clinical competence in the univariate analyses. Categorical variables were dummy-coded before entry into the regression model. The results of the analysis of factors associated with clinical competence are presented in Table 4. The assumptions of the regression model and multicollinearity were assessed. The Durbin–Watson statistic was 1.91, which is close to 2, indicating no substantial autocorrelation among the error terms. Tolerance values ranged from 0.37 to 0.97, exceeding the recommended threshold of 0.10, and variance inflation factor values ranged from 1.03 to 2.71, below the cutoff value of 10, indicating no evidence of multicollinearity. Therefore, the assumptions related to autocorrelation and multicollinearity for the regression analysis were met.

The multiple regression analysis showed that satisfaction with clinical practice, critical thinking disposition, AI literacy, and academic year were significantly associated with clinical competence. Specifically, students who were satisfied with clinical practice had higher clinical competence than those who were dissatisfied (β = 0.35, *p* < 0.001). Critical thinking disposition (β = 0.34, *p* < 0.001), AI literacy (β = 0.23, *p* = 0.001), and academic year, specifically being in the fourth year compared with the third year (β = 0.15, *p* = 0.009), were also significantly associated with clinical competence. The model explained 40% of the variance in clinical competence (R^2^ = 0.40, adjusted R^2^ = 0.39), and the regression equation was statistically significant (F = 26.11, *p* < 0.001).

## 4. Discussion

This study examined the associations of AI literacy and critical thinking disposition with clinical competence among nursing students with clinical practice experience and aimed to provide preliminary evidence regarding the educational relevance of AI literacy in nursing education.

The findings showed that clinical competence differed significantly according to academic year and satisfaction with clinical practice. In particular, fourth-year students had significantly higher levels of clinical competence than third-year students, consistent with the results reported by Jun [32]. This finding is also broadly consistent with an international study conducted in the Netherlands, in which fourth-year nursing students demonstrated significantly higher levels of clinical reasoning than second- and third-year students [38]. These differences may reflect the greater accumulation of nursing knowledge and clinical practice experience among students in more advanced academic years.

In addition, this study found that higher satisfaction with clinical practice was significantly associated with higher clinical competence among nursing students. This finding is consistent with the results of Kim [33], who reported that students who were satisfied with clinical practice demonstrated significantly higher clinical competence. Although the variables examined were not directly equivalent, a multi-country study of graduating nursing students also reported that positive perceptions of the clinical learning environment were associated with both higher self-assessed competence and greater satisfaction with the nursing education program and clinical practicum [39]. Students with higher satisfaction with clinical practice may perceive their clinical learning experiences more positively and participate more actively in nursing tasks, which may be associated with higher levels of clinical competence. Conversely, students with higher clinical competence may also report greater satisfaction with clinical practice. Taken together, these findings suggest that academic year and satisfaction with clinical practice may be considered when developing educational strategies related to clinical competence. In particular, learner-centered and supportive approaches may be considered for students at different academic levels.

Clinical competence did not differ significantly according to the frequency of generative AI use, purpose of generative AI use, or perceived reliance on generative AI. Among the participants, 63.0% reported using generative AI frequently, 72.0% reported using it primarily for assignments and studying, and 58.5% reported high perceived reliance on generative AI. These findings indicate that generative AI may already be widely used as a learning tool among nursing students and that responses regarding frequency of use, purpose of use, and perceived reliance were concentrated in particular categories. Therefore, these generative AI use-related characteristics alone may have had limited ability to distinguish differences in clinical competence.

The mean AI literacy score was 3.45 out of 5, which was slightly lower than the overall mean of 3.73 reported by Kang [13] but similar to the converted undergraduate mean of 3.48 in that study. The mean critical thinking disposition score was 3.74, slightly higher than the scores of 3.57 and 3.65 reported by Yang [40] and Heo [17], respectively, while the mean clinical competence score was 3.98, exceeding the scores of 3.30 and 3.60 reported by Lee [41] and Je [42]. These differences may be related to variations in participants’ academic level, curriculum, clinical practice experience, and timing of data collection, as well as the increasing use of active learning approaches and greater exposure to digital and AI-based learning environments in contemporary nursing education. Direct comparison with international studies was limited because few studies have examined comparable nursing student populations using the same instruments, particularly the AI literacy instrument. Therefore, these differences in mean scores should be interpreted cautiously.

AI literacy and critical thinking disposition showed a moderate-to-strong positive correlation. Although critical thinking disposition in the present study is not directly equivalent to critical thinking ability examined in previous research, this finding is broadly consistent with the multicenter study by Cui and Zhang [20], which found that AI literacy was positively associated with critical thinking ability among nursing interns and partially mediated the association between AI dependence and critical thinking ability. Taken together, these findings suggest that AI literacy and critical thinking disposition may be related and potentially complementary competencies in nursing education.

Critical thinking disposition and clinical competence showed a moderate positive correlation. This finding supports the results of Park [16], who reported that a higher critical thinking disposition was associated with higher clinical competence among nursing students. Although the participants in the international studies were clinical nurses rather than nursing students, similar associations have been reported. Tajvidi and Moghimi Hanjani [18] found that critical thinking disposition was positively correlated with clinical competence and significantly predicted clinical competence among Iranian nurses. Similarly, Chang et al. [19] reported a positive relationship between critical thinking ability and nursing competence among clinical nurses in Taiwan. This suggests that students with a stronger critical thinking disposition may be more likely to analyze complex clinical problems, determine priorities, and respond appropriately to clinical situations. At the same time, students with higher clinical competence may also report a stronger critical thinking disposition. Given this association, educational approaches aimed at fostering critical thinking dispositions among nursing students warrant further consideration.

AI literacy also showed a moderate positive correlation with clinical competence. Direct comparison was difficult because studies examining the relationship between AI literacy and clinical competence remain limited both domestically and internationally. However, previous research has shown that digital literacy is positively associated with clinical competence among nursing students [43]. In addition, the critical evaluation dimension of AI literacy may enable students to use AI-generated information appropriately while maintaining professional judgment, thereby supporting clinical reasoning and decision-making [25,28]. This may help explain the positive association between AI literacy and clinical competence observed in the present study. However, because the use of AI-generated information in clinical reasoning or decision-making was not directly assessed, this explanation requires further empirical examination.

According to the systematic review by Mousavi Baigi et al. [44], among 26 studies involving students in health-related disciplines, half reported low levels of AI-related knowledge. In addition, among 38 studies, 76% reported positive attitudes toward the use of AI, whereas 24% reported negative attitudes. Furthermore, in the study by Abuzaid et al. [45] involving nurses in the United Arab Emirates, 51% of respondents reported that they had acquired AI-related knowledge primarily through self-learning, and 75% indicated that basic AI education should be included in nursing curricula. Taken together, these previous findings suggest that, despite generally positive perceptions of AI, a lack of systematic educational experience has been consistently identified as a common issue. Given the increasing recognition of AI literacy as an important competency in healthcare and its observed association with clinical competence, systematic AI literacy education may be considered within formal nursing curricula. However, research examining this relationship remains limited, and future intervention studies are needed to determine whether such education can improve clinical competence.

In the final multiple regression analysis, satisfaction with clinical practice (β = 0.35), critical thinking disposition (β = 0.34), AI literacy (β = 0.23), and academic year (β = 0.15) were significantly associated with clinical competence. Notably, AI literacy remained significantly associated with clinical competence even after critical thinking disposition and clinical practice-related characteristics were considered. This finding suggests that AI literacy may be considered an additional competency associated with clinical competence in increasingly digitalized healthcare environments. At the same time, the relatively stronger associations of satisfaction with clinical practice and critical thinking disposition, compared with AI literacy and academic year, underscore the continuing importance of supportive clinical learning experiences and critical thinking competence. The regression model was statistically significant (F = 26.11, *p* < 0.001) and explained 40% of the variance in clinical competence (R^2^ = 0.40, adjusted R^2^ = 0.39), indicating that additional educational, psychological, and clinical factors not included in the model may also be associated with clinical competence.

This study is significant in that it examined the association between AI literacy and clinical competence among nursing students with clinical practice experience, an area that remains underexplored despite the rapid expansion of AI use in healthcare. In addition, by examining AI literacy and critical thinking disposition alongside academic year and satisfaction with clinical practice, this study provided a more comprehensive assessment of factors associated with clinical competence among nursing students.

This study has several limitations. First, its cross-sectional design precludes conclusions about temporal order or causality, and the observed associations may be bidirectional. Longitudinal and intervention studies are needed to clarify the direction of these relationships. Second, all key variables were measured using self-report questionnaires at a single time point, which may have introduced common method bias and affected the magnitude of the observed associations. In particular, self-reported clinical competence reflects perceived rather than objectively observed performance and may overestimate or underestimate actual competence. Future studies should incorporate objective or performance-based measures, such as OSCE scores, direct observation, and evaluations by instructors or clinical preceptors. Third, participants were recruited through convenience sampling from nine nursing colleges in a limited geographic region of the Republic of Korea, and most were female. Therefore, the findings may not be generalizable to nursing students in other regions, male nursing students, or students in different cultural, educational, technological, and healthcare contexts. Multicenter studies using more diverse and probability-based samples are needed. Fourth, residual confounding cannot be ruled out because some potentially relevant educational, psychological, and clinical factors were not included in the final model. Finally, the use of both face-to-face and online surveys may have introduced differences in response environments.

## 5. Conclusions

This study found that AI literacy and critical thinking disposition were significantly associated with clinical competence among nursing students with clinical practice experience. Satisfaction with clinical practice and academic year were also significantly associated with clinical competence in the final regression model.

This study adds to the limited evidence regarding the relationship between AI literacy and clinical competence in nursing education. The findings suggest that AI literacy may be an educationally relevant competency associated with clinical competence in increasingly digitalized healthcare environments. Accordingly, nursing education may consider programs and strategies that address AI literacy alongside critical thinking disposition.

## Figures and Tables

**Table 1:** Differences in clinical competence by general characteristics (*N* = 200).

Characteristics	Categories	*n* (%) or M ± SD (Range)	Clinical Competence (M ± SD)	t/F	*p*	Scheffé
Age (yrs)		22.18 ± 2.05 (20–33)				
Gender	Male	33 (16.5)	179.88 ± 25.39	0.19	0.852	
	Female	167 (83.5)	179.05 ± 22.70			
Academic year	3 ^a^	112 (56.0)	174.90 ± 23.62	−3.02	0.003	a < b
	4 ^b^	88 (44.0)	184.65 ± 21.33			
Satisfaction with clinical practice	Satisfied ^a^	86 (43.0)	187.53 ± 22.07	14.86	<0.001	c < b < a
	Moderate ^b^	89 (44.5)	175.80 ± 20.49			
	Dissatisfied ^c^	25 (12.5)	162.56 ± 24.12			
Academic performance	High	58 (29.0)	185.10 ± 21.81	2.78	0.064	
	Moderate	107 (53.5)	177.15 ± 23.71			
	Low	35 (17.5)	175.63 ± 22.11			
Frequency of generative AI use	Frequently	126 (63.0)	178.78 ± 21.89	0.24	0.786	
	Only when necessary	70 (35.0)	180.29 ± 24.74			
	Rarely	4 (2.0)	173.00 ± 35.67			
Purpose of generative AI use	Assignments and study	144 (72.0)	178.10 ± 22.54	0.91	0.462	
	Information summarization	21 (10.5)	183.81 ± 23.88			
	Information retrieval	31 (15.5)	181.87 ± 25.43			
	Entertainment, interaction, and others	4 (2.0)	173.25 ± 24.20			
Perceived reliance on generative AI	High	117 (58.5)	180.32 ± 20.44	0.71	0.494	
	Moderate	70 (35.0)	176.66 ± 27.19			
	Low	13 (6.5)	182.69 ± 22.30			

Note: AI = artificial intelligence; M = mean; SD = standard deviation; yrs = years. Lowercase letters indicate groups used for post hoc comparisons based on Scheffé’s test.

**Table 2:** Scores for AI literacy, critical thinking disposition, and clinical competence (*N* = 200).

Variables	M ± SD	Min–Max
AI literacy	65.51 ± 10.69	19~92
Critical thinking disposition	100.98 ± 11.23	65~133
Clinical competence	179.19 ± 23.10	107~225

Note: AI = artificial intelligence; M = mean; SD = standard deviation; Min = minimum value; Max = maximum value.

**Table 3:** Correlations among AI literacy, critical thinking disposition, and clinical competence (*N* = 200).

Variables	1	2	3
1. AI literacy	1		
2. Critical thinking disposition	0.59 ***	1	
3. Clinical competence	0.48 ***	0.51 ***	1

Note: AI = artificial intelligence; *** *p* < 0.001.

**Table 4:** Factors associated with clinical competence (*N* = 200).

Variables	B	S.E.	β	t	*p*
(Constant)	63.94	11.85		5.39	<0.001
AI literacy	0.50	0.15	0.23	3.34	0.001
Critical thinking disposition	0.69	0.14	0.34	4.87	<0.001
Academic year: 4th year	6.88	2.62	0.15	2.63	0.009
Satisfaction with clinical practice: satisfied	16.08	4.25	0.35	3.78	<0.001
Satisfaction with clinical practice: moderate	6.53	4.19	0.14	1.56	0.120
R^2^	0.40				
Adjusted R^2^	0.39				
F (*p*)	26.11 (<0.001)				

Note: AI = artificial intelligence; B = unstandardized regression coefficient; S.E. = standard error; β = standardized regression coefficient; R^2^ = coefficient of determination; reference categories for dummy variables: academic year = 3rd year; satisfaction with clinical practice = dissatisfied.

## Data Availability

Due to ethical and privacy restrictions related to human participant data and the need to protect participant confidentiality, the data presented in this study are available from the corresponding author upon reasonable request.

## Public Involvement Statement

There was no public involvement in any aspect of this research.

## Guidelines and Standards Statement

This manuscript was prepared in accordance with the STROBE (Strengthening the Reporting of Observational Studies in Epidemiology) reporting guidelines for cross-sectional studies.
